# LncRNAs in TGF-β-Driven Tissue Fibrosis

**DOI:** 10.3390/ncrna4040026

**Published:** 2018-10-04

**Authors:** Patrick Ming-Kuen Tang, Ying-Ying Zhang, Hui-Yao Lan

**Affiliations:** 1 Department of Medicine and Therapeutics, Li Ka Shing Institute of Health Sciences, Lui Che Woo Institute of Innovative Medicine, Shenzhen Research Institute, The Chinese University of Hong Kong, Hong Kong 999077, China; 2 Department of Anatomical and Cellular Pathology, State Key Laboratory of Translational Oncology, The Chinese University of Hong Kong, Hong Kong 999077, China; patrick.tang@cuhk.edu.hk; 3 Department of Nephrology, Tongji University School of Medicine, Shanghai 200065, China; idklaa@hotmail.com

**Keywords:** long noncoding RNA, fibrosis, transforming growth factor-β

## Abstract

Transforming growth factor-β (TGF-β) is a crucial mediator in tissue fibrosis that promotes accumulation of extracellular matrix (ECM), myofibroblasts to epithelial–mesenchymal transition (EMT), endothelial-mesenchymal transition (EndoMT), and apoptosis via canonical and noncanonical signaling pathways. In the past decades, a number of microRNAs have been reported to participate in TGF-β-mediated tissue scarring; however, the roles of long noncoding RNAs (lncRNAs) in fibrogenesis remain largely unknown. Recently, emerging evidence has shown that lncRNAs are involved in the development of different diseases, including cancer, autoimmune diseases, cardiovascular diseases, and fibrotic diseases. In this review, we summarize the current updates of lncRNAs in TGF-β1-driven tissue fibrosis and discuss their therapeutic potential for the treatment of chronic fibrotic diseases.

## 1. Introduction

Tissue fibrosis is one of the main pathogenic mechanisms for end stage organ diseases (e.g., chronic kidney disease, liver cirrhosis, congestive heart failure) that lead to high morbidity and increase health care burden worldwide [1]. Understanding the underlying molecular mechanisms of tissue fibrosis would help to identify effective therapeutic targets for controlling chronic diseases. Emerging evidence shows that noncoding RNAs (ncRNAs), including microRNAs (miRNAs), short interfering RNAs (siRNAs), piwi-interacting RNAs (piRNAs), and various types of long ncRNAs (lncRNAs) [2] may be involved in the development and progression of fibrotic diseases [3,4]. In the last decade, the working mechanisms of miRNAs have been identified in many chronic diseases, including kidney disease, pulmonary fibrosis, and hepatic fibrosis [5,6]. However, the development of miRNA-based therapy was largely limited by their low tissue- and organ-specificity in transcriptional regulation [7]. By contrast, the therapeutic potential of lncRNAs, a class of RNAs longer than 200 nucleotides without protein coding capacity, has started to be considered nowadays [8,9].

Transforming growth factor-β (TGF-β) has been reported as a crucial profibrotic cytokine that triggers fibrogenesis in a number of chronic diseases, especially chronic kidney disease (CKD) [10,11]. Previous studies showed that TGF-β1 can induce renal fibrosis via both canonical (Smad-dependent) and noncanonical (Smad-independent) signaling pathways, which results in a serial of fibrotic events, including excessive accumulation of extracellular matrix (ECM), inhibition of ECM degradation, induction of fibroblast proliferation, and myofibroblasts to epithelial–mesenchymal transition (EMT) [12,13,14,15]. In this review, current findings regarding TGF-β1-driven fibrosis via lncRNAs and their therapeutic perspectives in organ fibrosis will be discussed in detail.

### 1.1. Transforming Growth Factor-β1 Signaling during Fibrogenesis

TGF-β is well-known as a cluster of secreted signaling proteins, which was named after the discovery of its first member in 1983 [16]. The TGF-β superfamily consists of four major subfamilies: The TGF-β subfamily, the bone morphogenetic proteins (BMPs), the activin and inhibin subfamilies, and a group encompassing various divergent members [17]. TGF-β1, TGF-β2, and TGF-β3 are three distinct isoforms, which have been extensively found in mammal tissues. TGF-β1 lacks a TATAA box in its promoter region and is mainly regulated via AP-1 sites. By contrast, both of the TGF-β2 and TGF-β3 promoters contain TATAA boxes and AP-2 sites which are regulated by the cAMP-responsive elements [18]. TGF-β1 is well known as a key profibrotic mediator in fibrotic diseases due to the distinctive promoter region from TGF-β2 and 3. TGF-β1 gene expression is activated directly by transactivation proteins, exerting a mechanistic basis for wound healing, tissue repair, pathogenesis of viral-mediated disease, and organ fibrogenesis, while also acting as a major regulator of cellular growth and differentiation [19].

TGF-β1 has diverse roles under pathological conditions. In mice, targeted disruption of the *TGF-β1* gene led to the development of autoimmune disease [20]. TGF-β1-knockout mice develop systemic inflammation, resulting in lymphocytic perivasculitis, plasmacytic infiltration, and interstitial pneumonia [21]. Disruption of TGF-β1 expression in the lung epithelium affects lung morphogenesis and epithelial differentiation [22]. Overexpression of hepatic TGF-β1 causes severe renal damage in mice with progressive renal fibrosis [23]. Indeed, TGF-β1 is essential for the development of liver diseases, especially in the inflammation-driven liver cirrhosis. In normal hepatic physiology, sinusoidal endothelial cells and Kupffer cells (macrophages) express high levels of TGF-β1, in contrast to hepatic stellate cells (HSC), which do not express TGF-β1 when quiescent [24]. However, during fibrogenesis, HSC change to produce all isoforms of TGF-β in the injured liver, especially a high level of TGF-β1 for activating myofibroblasts and triggering massive hepatocyte cell death [25]. Importantly, inhibition of TGF-β1 or its downstream signaling showed therapeutic effects on tissue fibrosis in a wide range of experimental disease models.

TGF-β1 has been demonstrated to be one of the major pathogenic promoters in the development of organ fibrosis, as mentioned above [10,19,26,27]. TGF-β1 transduces its signal by binding and bringing together two single-pass transmembrane receptor kinases into a kinase-active complex. TGF-β1 covalently binds the latency-associated peptide (LAP) via disulfide bonds in the endoplasmic reticulum and forms as an inactive complex as latent TGF-β binding proteins (LTBP). The latent TGF-β1 complex is then cleaved by a wide range of proteases to release the active TGF-β1, which bind to the extracellular domain of TGF-β receptor type II (TβRII) that further activates TGF-β receptor type I (TβRI) kinase, which triggers downstream signaling to exert its biological functions [28]. As noted, TGF-β1 signaling can exert its biological functions via both canonical and noncanonical pathways.

**Figure 1:**
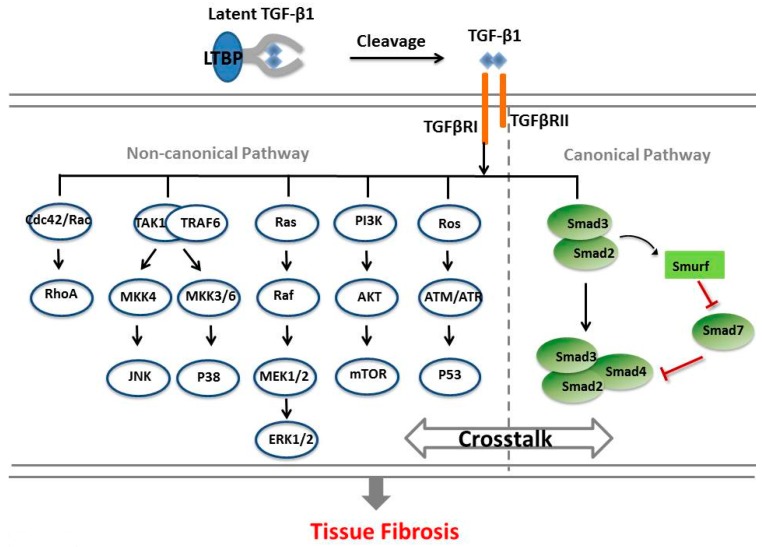
Transforming growth factor-β 1 (TGF-β1) mediates a signaling pathway in tissue fibrosis. The latent TGF-β binding proteins (LTBP) complex is cleaved by proteases to release the active TGF-β1 that binds to the extracellular domain of TGF-β receptor type II (TβRII). The activated TβRII then phosphorylates TGF-β receptor type I (TβRI) kinase, thus triggering downstream signaling via either or both of the canonical (Smads-dependent) and noncanonical (Smads-independent) pathways. In the canonical pathway, TβRI phosphorylates Smad2 and Smad3, and then these Smads bind with Smad4 and this complex translocates into the nucleus. Meanwhile, TGF-β1 also activates Smad ubiquitin regulatory factor (Smurf) to degrade Smad7 to further enhance signaling. On the other hand, TGF-β1 can also induce profibrotic responses via a noncanonical pathway in a Smads-independent manner. TGF-β1 activates extracellular signal-regulated kinase (ERK) activation (Ras recruits Raf to the plasma membrane and leads to activation of ERK through mitogen-activated protein kinase (MEK)); c-Jun amino terminal kinase (JNK)/p38 activation JNK and p38 are at the tertiary layer of the mitogen-activated protein kinase (MAPK) pathway, in which they are activated by the MAP kinase kinases (MKKs), MKK4 and MKK3/6, respectively); Rho-like GTPases activation (the Rho-like GTPases include RhoA, Rac, and Cdc42); Phosphoinositide3-kinase/RAC-alpha serine/threonine-protein kinase (PI3K/AKT) activation (AKT is activated via PI3K, which then controls translational responses through mammalian target of rapamycin (mTOR)); induction of reactive oxygen species (ROS) (hypoxia-responsive element activity and hypoxia-inducible factor-1α expression by TGF-β1, then the p53 tumor suppressor can be induced). In addition, crosstalks may occur between TGF-β1/Smad and other pathways during tissue fibrosis.

TGF-β1 activates a canonical signaling pathway that involves the participation of Smad proteins, Smad2, Smad3, and Smad4, which exert distinct and even opposing functions during the process of fibrotic regulation [29,30]. Activation of TβRI induces phosphorylation of Smad2 and Smad3, leading to the formation of a complex of these two proteins with Smad4, which then translocates to the nucleus [31]. Meanwhile, TGF-β1 activates a Smad ubiquitin regulatory factor (Smurf) that directly degrades Smad7 (a Smad3 inhibitory protein) via post-transcriptional modification, and thereby further enhances TGF-β1/Smad signaling [32]. In the canonical TGF-β1 signaling pathway, the most important pathogenic transcription factor is Smad3. A number of studies demonstrated that targeting Smad3 can ameliorate the development and progression of tissue fibrosis in vivo, including experimental models of renal fibrosis, pulmonary fibrosis, and liver fibrosis [13,33,34,35,36]. In other words, the imbalance of the Smad proteins (Smad2/Smad3 and Smad7) is the key TGF-β1-dependent mechanism for promoting tissue fibrosis. Details are summarized in Figure 1.

In addition, TGF-β1 can induce fibrotic response through a noncanonical pathway in a Smad-independent manner. TGF-β1 can activate the mitogen-activated protein kinase (MAPK) pathway via extracellular signal-regulated kinase (ERK), p38/MAPK, and c-Jun-N-terminal kinase (JNK), as well as directly trigger the TGF-β1 activates kinase 1, phosphatidylinositol 3 kinase/Akt, Abelson nonreceptor tyrosine kinase(c-Abl), and Rho GTPase pathways [37] (Figure 1). TGF-β1 can also induce apoptosis of endothelial cells and podocytes and promote renal fibrosis [38]. TGF-β1 is a potent inducer of a mesenchymal gene expression program that induces the transition of epithelial cells, endothelial cells, and intrinsic renal fibroblasts into α-smooth muscle actin (SMA)-expressing myofibroblasts, referred to as EMT and endothelial–mesenchymal transition (EndoMT) [15].

Collectively, TGF-β1 accelerates renal fibrosis through various mechanisms by causing cell loss through apoptosis, creating imbalance in fibroblast-mediated ECM synthesis and ECM accumulation, and inducing the transition of various cell types, including epithelial cell, endothelial cells, and macrophages into fibroblast-type cells that are capable of depositing ECM [36,39,40,41].

### 1.2. The Outline of Long noncodingRNAs

In the highly dynamic genome, only about 2.2% of the total genes are under conservation constraints [42]. Among them, noncoding genes are poorly conserved, where more than 80% of lncRNAs are of primate origin [43]. Hezroni et al. analyzed noncoding transcriptome profiles of 17 different species (16 vertebrates and a sea urchin) and concluded that most of the full sequence of noncoding genes tends to be conserved, although only the short patches of conserved sequences found at their 5′ ends, which effectively preserve the expression patterns of lncRNAs in species, especially those involved in evolution [44]. Classification of lncRNAs is based on not only the length, properties, and genomic location of the transcripts, but also their genomic annotation, regulatory element, and function [45]. With respect to length, these noncoding RNAs can be categorized into lncRNAs, very long intergenic ncRNAs (vlincRNAs), and macro lncRNAs [46,47,48]. According to their genomic location within protein-coding gene (PCG), lncRNAs can be further classified as intergenic, intronic, antisense, or overlapping sense transcripts [49,50], as well as based on residence within specific DNA regulatory elements and loci, function, and association with specific biological processes [51].

Interestingly, there are more than 98% genes unexpectedly identified as noncoding RNA, but only about 1.2% of genes encode proteins [52]. The molecular functions of lncRNAs are dependent on their archetypes, which facilitate their actions in signals, decoys, guides, or scaffolds [53]. They execute their biological effects mainly via transcriptional or post-transcriptional gene regulation by affecting chromatin structure, RNA maturation, protein synthesis, and transport [54].

The first reported regulatory ncRNA was found in bacteria in the 1970s, and then large amounts of ncRNAs were identified from the eukaryotic organisms [2]. Since the last decade, a number of newly discovered lncRNAs have been fully characterized, such as H19 and Xist [55,56]. Interestingly, lncRNAs are predicted to be largely expressed at a level that exceeds that of protein-coding transcripts, but their biological functions and regulatory mechanisms still remain largely unknown and hotly debated. Indeed, lncRNAs have a remarkably strong conservation expression throughout biological evolution, suggesting that they are selectively maintained and crucial for developmental processes.

### 1.3. Long Noncoding RNAs in the Regulation of Transforming Growth Factor-β1/Smad Signaling

There were 21 Smad3-dependent novel lncRNAs discovered from two kidney injury mouse models with unilateral ureteral obstructive (UUO) nephropathy and immunologically induced antiglomerular basement membrane glomerulonephritis using high throughput RNA sequencing in our previous work. These novel lncRNAs were Smad3-dependently mediated and may contribute to the renal inflammation and fibrosis in the injured kidneys, which correlated with the progression of experimental kidney diseases in mice [57]. Furthermore, the biological roles of several novel lncRNAs from these 21 Smad3-dependent lncRNAs in kidney diseases were intensively elucidated. Arid2-IR (np 28496), a Smad3-associated lncRNA, is named due to the fact that its location is overlapped with a protein-coding gene Arid2-IR. The function of Arid2-IR has been identified as to promote NF-κB-dependent renal inflammation in kidney fibrosis [58]. Furthermore, another novel Smad3-dependent lncRNA Erbb4-IR (np 5318) has been demonstrated to promote renal fibrosis in both UUO-induced and diabetic nephropathy mouse models through suppressing Smad7 and miR-29b, respectively; targeting renal Erbb4-IR effectively inhibited the progression of kidney fibrosis in mice [59,60]. In addition, some other groups also detected Smad3-associated lncRNAs. Sun et al. analyzed the renal and urinal transcriptome profiles of the UUO and sham-operated rats, and identified 103 disease-associated lncRNAs, including 24 up-regulated and 79 down-regulated lncRNAs in the fibrotic kidney of a rat UUO model. Mechanistic study further revealed that some of the lncRNAs contain potential binding sites for Smad3, and eventually demonstrated that two novel lncRNAs, TCONS_00088786 and TCONS_01496394, might be critically expressed during renal fibrogenesis due to their regulatory role in TGF-β/Smad signaling and the transcriptional feedback mechanism [61].

In addition to renal fibrosis, lncRNAs are involved in the regulation of the TGF-β/Smad canonical pathway in the development of tissue fibrosis in other important organs. The expression of lncRNA H19 is significantly up-regulated in idiopathic pulmonary fibrosis in a bleomycin-(BLM) induced lung fibrosis model and leads to ECM deposition in vivo and in vitro [62]. LncRNA PFRL (NONMMUT022554), a novel lncRNA, was found to be increased in fibrotic lung tissues of mice and pulmonary fibroblasts under TGF-β1 stimulation in vivo and in vitro [63]. Further study identified the pathogenic role of lncRNA PFRL in lung fibrosis, which acts via modulation of a miR-26a/Smad2 feedback loop. Fu and colleagues showed that lncRNA-ATB (lncRNA-activated by transforming growth factor-beta) is a key regulator involved in TGF-β-induced liver cirrhosis and vascular invasion of hepatocellular carcinoma [64]. LncRNA-ATB was found to share the common miRNA responsive element of miR-425-5p with TGF-βRII and Smad2, and TGF-β up-regulated lncRNA-ATB expression via competitive binding to miR-425-5p, leading to collagen I production in activated HSCs during HCV-induced liver fibrogenesis [65]. Zhang et al. reported a liver-enriched lncRNA named liver fibrosis-associated lncRNA1 (lnc-LFAR1); mechanistic studies revealed that lnc-LFAR1 promotes liver fibrosis by directly regulating the binding of Smad2/3 to TGFβR1 that develops a TGF-β1/Smad2/3/lnc-LFAR1 feedback loop [66].

### 1.4. Long Noncoding RNAs in Transforming Growth Factor-β1-Induced Extracellular Matrix Accumulation

Tissue fibrosis is a debilitating condition which occurs during end stage organ failure, where excessive accumulation of ECM proteins is one of the important pathogenic processes. Indeed, TGF-β1 is a potent driver for triggering ECM production in fibrosing tissues [67], where the involvement of lncRNAs has been recently revealed.

For liver fibrosis, Zhou et al. identified over 3600 lncRNAs that are expressed in human HSC myofibroblasts. Many of these are involved in the key fibrotic signaling and networking with ECM-related genes for tissue scarring by the regulation from TGF-β [68]. Co-expression analyses were applied to identify the potential function of noncoding genes on the protein-coding genes that regulate TGF-β-induced ECM accumulation. Finally, 12 lncRNAs were identified related to ECM production and liver fibrosis by performing gene ontology (GO) enrichment analysis. LncRNA maternally expressed gene 3 (lncRNA MEG3) was found to be down-regulated in both an experimental carbon tetrachloride (CCl4)-induced model of liver fibrosis and fibrotic patients; overexpression of MEG3 decreased HSC activation by suppressing ECM protein synthesis [69].

For cardiac fibrosis, Huang et al. demonstrated that the expression of lncRNAs was dynamically regulated in ischemic cardiomyopathy (ICM), where some lncRNAs also participated in the TGF-β pathway to promote expression of genes related to ECM accumulation and myofibroblast differentiation. An analysis of the lncRNA–mRNA expression correlation coefficient matrix between 145 differentially expressed lncRNAs and 285 differentially expressed mRNAs identified several lncRNAs showing strong positive expression correlation with protein-coding genes related to ECM, such as lncRNA n379599 and n342359, which were increased in ICM hearts. Furthermore, they analyzed the functional annotation of this lncRNAs–mRNA expression correlation using bioinformatic platform Database for Annotation, Visualization and Integrated Discovery (DAVID), and found the candidates were dominated by the collagen and ECM protein encoding genes (e.g., COL14A1, COL16A1, COL12A1, COL8A1) [70].

For renal fibrosis, the lncRNA plasmacytoma variant translocation 1 gene (*PVT1*) was found to be highly expressed in a variety of renal cell types [71]. Knockdown of *PVT1* significantly reduced mRNA and protein levels of FN and COL4A1, as well as TGF-β1 and PAI-1, suggesting that PVT1 might affect ECM proteins production in renal fibrosis [71].

### 1.5. Long Noncoding RNAs in Transforming Growth Factor-β1-Driven Epithelial-Mesenchymal Transition

EMT characterized by the progressive loss of cell-to-cell contacts modulates cell polarity, rearranges cytoskeletons, and intermediates filament switch from the typical cytokeratins into vimentin [72,73,74]. EMT is a key biological process involved in a number of developmental and pathological events, including fibrogenesis [75]. The role of TGF-β1 as an EMT-inducer has not been fully studied: The molecular mechanisms regulating this transition and their implications in fibrosis are still largely unexplored. TGF-β signaling regulates the expression and activity of transcription factors that elicit EMT through direct and indirect mechanisms. TGF-β1 is reported to induce expression of ligands for many other pathways, such as RTK signaling, β-catenin signaling, and Notch signaling [76]. TGF-β1-induced EMT is enhanced due to its key role in the process of transcriptional and post-transcriptional mechanisms, and emerging evidence underlines the critical roles of lncRNAs in these processes [77].

The pathogenic role of lncRNA H19 (H19) has been elucidated in a number of inflammatory and organ fibrosis diseases, such as osteoarthritis, ulcerative colitis, liver fibrosis, renal fibrosis, and pulmonary fibrosis [78,79,80]. Yang et al. showed that H19 regulates TGF-β1-induced EMT via the PI3K/AKT pathway in vitro [81]. LncRNA-NR_033515 significantly increased in the serum of diabetic nephropathy (DN) patients and was related to the progression of diabetic nephropathy. Overexpression of NR_033515 accelerated EMT induced by TGF-β1 via regulating P38, ASK1, fibronectin, α-SMA, E-cadherin, and vimentin expressions by miR-743b-5p [82]. LncRNA HOTAIR increased in both UUO rats and TGF-β1-induced HK-2 cells in vivo and in vitro, whereas further mechanism investigation revealed that HOTAIR promotes EMT by down-regulating miR-124 to enhance the Notch1 pathway [83].

Recently, Liu et al. applied microarray and experimental data to systematically examine ncRNA co-expression profiles in human alveolar epithelial cells [84]. They found that 33 lncRNAs act as competing endogenous RNAs (ceRNAs) for miRNAs and play a regulatory role in the physiological and pathological processes during TGF-β1-induced EMT. Another study utilized online public bioinformation from both UCSC genome online (http://genome.ucsc.edu/) and NCBI database (http://blast.ncbi.nlm.nih.gov/), and identified two lncRNAs, uc. 77 and 2700086A05Rik (05Rik), which overlap with genes encoding two important EMT regulators, Zeb2 and Hoxa3 [85,86]. Zinc-finger enhancer binding 2 (Zeb2) is a critical transcription factor subjected to the initiation of EMT, and Hoxa3 belongs to the homeobox family of genes which encode a highly conserved family of transcription factors that are related to morphogenesis and cell differentiation. The study suggested that Zeb2 and Hoxa3 might be involved in EMT in pulmonary fibrosis [84,87].

### 1.6. Long Noncoding RNAs in Other Transforming Growth Factor-β1-Dependent Fibrotic Mechanisms

TGF-β1 also triggers tissue scarring via inducing EndoMT in the damaged organ [88]. Blocking TGF-β1 signaling resulted in reduced EndoMT and neointimal formation in a mouse vein graft model [89]. The metastasis-associated lung adenocarcinoma transcript 1 (MALAT1) has been found to be induced in a TGF-β1-dependent manner; it facilitates TGF-β1-triggered EMT in retinal pigment epithelial cells that further enhance EndoMT via suppressing miR-145 expression in vitro [90].

TGF-β1 can also induce apoptosis of endothelial cells, thereby promoting tissue fibrosis. LncRNA AC067945.2 is decreased in hypertrophic scar tissues. Overexpression of AC067945.2 did not affect cell proliferation, but mildly promoted early apoptosis in normal skin fibroblasts [91]. TGF-β-mediated methylation of MEG3 promoter causes a decrease in the expression of this antifibrotic lncRNA, which accelerates liver fibrosis [69]; meanwhile, MEG3 could induce lncRNA p53-mediated apoptosis via modulating Bax/Bcl-2 and cytoplasmic cytochrome expression [92].

Furthermore, TGF-β1 could trigger fibrogenesis by acting directly on the tissue resident fibroblasts. Zhou et al. proposed that lncRNAs might also play a key role in cell identity and investigated whether TGF-β signaling directly regulates expression of lncRNAs in HSC myofibroblasts. Unexpectedly, they found that the lncRNAs expression may be solely regulated via their super-enhancers in the hepatic stellate cells (HSCs), rather than indirectly targeted by the TGF-β1 signaling, suggesting that TGF-β1 signaling mediates lncRNAs expression via super-enhancers instead of its direct regulatory mechanism on the protein-coding genes [68].

## 2. Therapy and Perspectives

Excessive TGF-β1 contributes to the development of tissue fibrosis that compromises normal organ function, and TGF-β1 blockade is one of the strategies to ameliorate the organ fibrosis in a number of animal disease models. However, TGF-β1 also functions as an anti-inflammatory cytokine in normal physiology [93]. Many studies demonstrated that targeting of TGF-β might potentially impair host immunity and cause autoimmune diseases [28]. Our group revealed that TGF-β1-deficient anti-GBM nephropathy mice developed lethal inflammation and death at 3 weeks of age [33,94]. Precision therapeutic strategies to inhibit TGF-β-mediated actions specific for disease development should be focused on the downstream profibrotic effector genes of TGF-β signaling. Thus, a number of TGF-β-mediated miRNAs and transcription factors have been identified [4]. For miRNAs, due to the complexity of regulatory and working mechanisms, different genes can be regulated by a single miRNA and vice versa. Furthermore, miRNAs may show the same pattern of expression, but some of the cluster members may give different expression patterns in different tissues. For Smads, Smad3 interacts with other signaling pathways (e.g., NF-kB) to regulate renal inflammation and fibrosis [95]. LncRNAs may serve as ceRNAs or molecular sponges for modulating miRNA expression and biological function. Indeed, emerging evidence demonstrated that lncRNAs are highly disease- and tissue-specific and, as such, may represent ideal therapeutic targets for fibrotic diseases. LncRNAs are far more specific and conserved to organs, tissues, cell types, and disease conditions as diagnostic and prognostic biomarkers, compared to protein-coding transcriptomes [96].

At present, the efficiency of tissue-specific lncRNAs-targeted therapy is being investigated in vivo and delivery strategies, including packaging strategies, receptor-mediated uptake, local activation, vector-based enrichment, and delivery by devices are being actively explored. Our group showed that silencing of Erbb4-IR in the kidney, using a non-invasive ultrasound microbubble-mediated technique, significantly improved renal function and inhibited renal fibrosis in both diabetic and UUO-injured mice [59,60]. In mice with idiopathic pulmonary fibrosis, knockdown of PFRL ablated BLM-induced pulmonary fibrosis [63]. In cardiac fibrosis, lentivirus mediated inhibition of lincRNA-p21 resulted in neointima hyperplasia in a carotid artery injury model, and siRNA-mediated silencing of lncRNA APF significantly reduced ischemia/reperfusion injury in mice [97,98].

However, challenges remain for developing effective lncRNAs-targeted therapy. For example, high concentrations and repeated dosing of siRNAs and gapmers are required to achieve sufficient inhibition of lncRNAs in vivo, but the potential dose-dependent toxicities might limit its therapeutic effects during preclinical and clinical testing. The toxicity observed is mainly classified into two types, hybridization-dependent and hybridization-independent toxicities. The former toxicity is due to the sequence of the oligonucleotide, while the latter one is due to the backbone and modifications of the oligonucleotide. In addition to the general toxicities of RNAs, gapmers might induce hepatotoxicity in an RNase H1-dependent manner. Optimizing the level of RNase H1 before gapmers treatment significantly prevents hepatotoxic events, indicating that hepatotoxicity of locked nucleic acid (LNA)-modified gapmers is good for RNase-dependent RNA degradation [99].

Another unresolved question involved in lncRNAs-targeted treatment is the fact that, unlike miRNAs, lncRNAs are relatively conserved among different species [100]. Unfortunately, there is still a lack of effective methods to identify lncRNA homologues amongst different species. Experimental methods to identify lncRNAs with therapeutic potential and clinical translational capacity from animal models are still in difficulty. Taken together, basic and preclinical research focusing on identifying and developing lncRNAs-based therapeutics is needed to translate bench findings into important rationales for the clinical setting.

## 3. Conclusions

The TGF-β1-driven fibrotic signaling pathway is dramatically activated in both experimental animal models and human end stage organ diseases. Because of the physiological importance of TGF-β1, we proposed to specifically cancel the profibrotic effects of the TGF-β1 pathway by identifying its downstream pathogenic regulators, including ncRNAs. With the development of ncRNA research, emerging evidence shows that lncRNAs play an important role in the development and progression of tissue fibrosis. Among the expanding interests in lncRNA research, we look forward to the continuous discovery of new regulatory and working mechanisms of lncRNAs in tissue fibrosis. Further understanding of the pathogenic mechanisms of lncRNAs will finally identify novel and effective targets for the development of therapeutic strategies for chronic diseases.
